# Adjuvant Chemotherapy, with or without Taxanes, in Early or Operable Breast Cancer: A Meta-Analysis of 19 Randomized Trials with 30698 Patients

**DOI:** 10.1371/journal.pone.0026946

**Published:** 2011-11-01

**Authors:** Ying-Yi Qin, Hui Li, Xiao-Jing Guo, Xiao-Fei Ye, Xin Wei, Yu-Hao Zhou, Xin-Ji Zhang, Chao Wang, Wei Qian, Jian Lu, Jia He

**Affiliations:** 1 Department of Health Statistics, Second Military Medical University, Shanghai, China; 2 Clinical Medicine, College of Basic Medicine, Shanghai Jiaotong University School of Medicine, Shanghai, China; Univesity of Texas Southwestern Medical Center at Dallas, United States of America

## Abstract

**Background:**

Taxanes have been extensively used as adjuvant chemotherapy for the treatment of early or operable breast cancer, particularly in high risk, node-negative breast cancer. Previous studies, however, have reported inconsistent findings regarding their clinical efficacy and safety. We investigated disease-free survival (DFS), overall survival (OS), and drug-related toxicities of taxanes by a systematic review and meta-analysis.

**Methodology and Principal Findings:**

We systematically searched PubMed, EMBASE, the Cochrane Center Register of Controlled Trials, proceedings of major meetings, and reference lists of articles for studies conducted between January 1980 and April 2011. Randomized controlled trials (RCTs) comparing chemotherapy with and without taxanes in the treatment of patients with early-stage or operable breast cancer were eligible for inclusion in our analysis. The primary endpoint was DFS. Nineteen RCTs including 30698 patients were identified, including 8426 recurrence events and 3803 deaths. Taxanes administration yielded a 17% reduction of hazard ratio (HR) for DFS (HR = 0.83, 95% CI 0.79–0.88, p<0.001) and a 17% reduction of HR for OS (HR = 0.83, 95% CI 0.77–0.90, p<0.001). For high risk, node-negative breast cancer, the pooled HR also favoured the taxane-based treatment arm over the taxane-free treatment arm (HR = 0.82, 95% CI 0.77–0.87, p = 0.022). A significantly increased rate of neutropenia, febrile neutropenia, fatigue, diarrhea, stomatitis, and oedema was observed in the taxane-based treatment arm.

**Conclusions/Significance:**

Adjuvant chemotherapy with taxanes could reduce the risk of cancer recurrence and death in patients with early or operable breast cancer, although the drug-related toxicities should be balanced. Furthermore, we also demonstrated that patients with high risk, node-negative breast cancer also benefited from taxanes therapy, a result that was not observed in previous studies.

## Introduction

Breast cancer (BC) is a leading cause of morbidity and mortality among women worldwide [1]–[2]. Most BCs (>75%) are diagnosed at an early stage or are operable [3]. For these patients, it is essential to administer adjuvant chemotherapy to reduce the risk of recurrence [4]–[5]. Taxanes(paclitaxel or docetaxel) are active cytotoxic agents that promote polymerization of tubulin and stabilization of microtubules by preventing their disassembly. Recently, several randomized trials have been conducted to identify the efficacy and safety of taxane-based adjuvant chemotherapy for early or operable BC, often with conflicting results. Additionally, the efficacy of taxanes for patients with high risk, node-negative BC remains uncertain. Two previous meta-analyses [6]–[7] have been conducted to determine the efficacy and safety of this agent in patients with BC although investigators did not present the efficacy of taxanes in node-negative BC. We undertook a meta-analysis to update the results and resolve the uncertain efficacy of taxanes in women with node-negative BC. Furthermore, we also reported the efficacy of taxanes treatment in some specific subgroups.

## Methods

### Search strategy and selection criteria

Randomized controlled trials (RCTs) and literatures trials resulted of taxane therapy were eligible for inclusion in our meta-analysis, with no restriction on language or publication status (i.e., published, unpublished, in press or in progress). The search process was initiated as follows:

1. Electronic databases (from January 1980 to April 2011): We retrieved literatures from PubMed, EmBase and the Cochrane Center Register of Controlled Trials, using the search terms of “early breast cancer,” “operable breast cancer,” “node-negative breast cancer,” “stage I or stage II breast cancer,” and “docetaxel or taxane or paclitaxel”.
2. Additional resources: Two important annual meetings including American Society of Clinical Oncology Annual Scientific Meeting (ASCO) and the San Antonio Breast Cancer Symposium (from 1995 to 2011), were manually searched. In addition, information about registered randomized controlled trials was obtained from the website http://clinicaltrials.gov/ (US NIH). Relevant reviews and meta-analyses regarding the role of taxane-based adjuvant chemotherapy in patients with early or operable BC were examined for potential trials.

This review was conducted and reported according to the PRISMA (Preferred Reporting Items for Systematic Reviews and Meta-Analysis) Statement issued in 2009 (Checklist S1) [8].

The eligible RCTs should meet the following inclusion criteria: (a) early or operable BC; (b) high quality RCT comparing a taxane-based adjuvant chemotherapy arm with a taxane-free adjuvant chemotherapy arm; and (c) the primary outcome was either disease-free survival (DFS) or overall survival (OS). Search and selection of studies was conducted independently by 2 investigators (Y-YQ and X-JG).

### Data extraction and quality assessment

Data extraction and quality assessment were conducted independently by 2 investigators (Y-YQ and HL) using a standardized data recording form and Jadad scale [9]. Information was examined and adjudicated independently by 2 additional investigators (X-FY and Y-HZ) referring to the original articles after data extraction and assessment.

The following information was extracted from each eligible study: study design, year of publication, number of patients, regimen details, median follow-up, median age, node status, main endpoint, the hazard ratios (HRs) and corresponding 95% confidence interval (CI), and the drug-related toxicities (WHO grades ≥3). For studies which reported HRs for the taxane-free treatment arm rather than the taxane-based treatment arm, HRs were recalculated by the exponential of negative ln(HR). If HRs and 95% CIs were not directly obtained from the original articles, they were estimated indirectly using reported events in each arm and the corresponding P value as suggested by Tierney et al [10]. If information could not be obtained from the original literature, direct communication with the authors was initiated. The quantitative 5-point Jadad scale was used as a gauge to assess the quality of the inclusive trials in our study.

### Statistical analysis

The primary efficacy outcome of our meta-analysis was disease-free survival (DFS). DFS was defined as time from randomization to any recurrence of BC (local or distant), new primary BC, a second cancer, or death. The subgroup analyses were prospectively planned according to node status, drug dosage, schedule, observation period, menopausal status, hormone receptor status, and tumor size. Interaction tests were performed to compare differences between the 2 estimates [11]. The adverse events (AEs) of taxane-based treatment were analyzed as drug-related toxicities (WHO grades ≥3). The pooled estimation plotted as odd ratios (ORs) was obtained [12]. A pooled OR and 95% CI greater than 1 indicated a statistically significant result.

Heterogeneity between trials was evaluated by chi-square (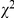) test and I-squared (I^2^) statistic [13]. These indices assess the percentage of variability across studies attributable to heterogeneity rather than chance. Statistical heterogeneity was considered significant when p<0.10 for the 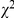 test or I^2^>50%. Although fixed-effects model and random-effects model yielded similar conclusions, we chose to use the random-effects model, which assumed that the true underlying effect varied among included trials. Moreover, many investigators consider that the random-effects model to be a more natural choice than fixed effects model in medical decision-making contexts [14]–[15]. The probability of publication bias was assessed with the funnel plots and the Begg-Mazumdar test [16]. Additionally, the pooled HR estimates were recalculated after excluding low-scoring trials to test their sensitivity. All reported P values were two-sided and P values less than 0.05 were regarded as statistically significant. Statistical analyses were carried out using STATA 11.0 (Stata Corporation, Lakeway, Texas, USA).

## Results

### Trial characteristic

**Figure 1:**
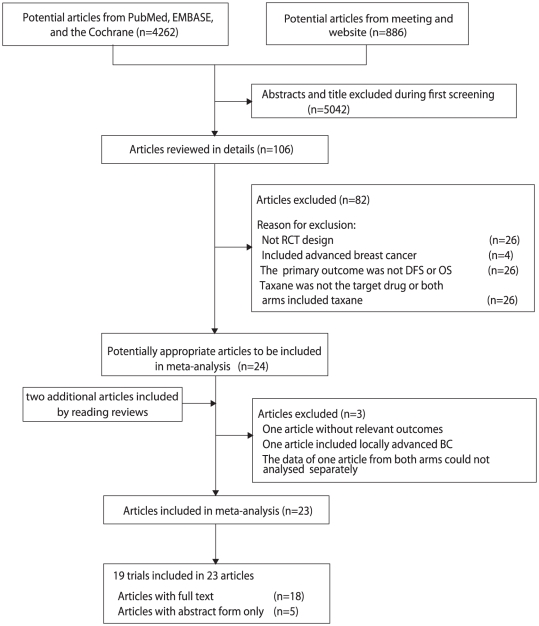
Flow diagram of the trials search and selection process.

Twenty-two potential trials were identified and 3 trials [17]–[19] of them were excluded for specific reasons listed in flow chart (Figure 1 and Protocol S1 [8]). The remaining 19 trials [20]–[42] included 30698 women with early or operable BC. Two trails [30]–[31], [33] were published in abstracts and the remaining 17 trials [20]–[27], [29], [32], [34]–[37], [39]–[42] were published in full articles. All of the trials included were open-label, phase III, randomized trials. Concurrent regimens were conducted in 5 trials [20], [22], [25], [27], [37], while sequential regimens were tested in the remaining 14 trials [21], [23]–[24], [29]–[30], [32]–[36], [38], [40]–[42]. The GEICAM 9805 [20] recruited patients with node-negative breast cancer, and the ECTO trial [38] only recruited patients with tumor size >2 cm. Recurrence/relapse-free survival (RFS) was the main endpoint of FinHer and Boccardo et al [23], [34], and freedom from progression (FFP) was the main endpoint of the ECTO trial [38]. However, the definition of RFS and FFP of these 3 trials was similar to DFS, so we included them. Fourteen [20]–[21], [23]–[24], [27], [29]–[30], [32], [34], [37]–[38], [40]–[42] of the 19 trials had Jadad scores of 3, and 5 trials [22], [26], [33], [35]–[36] were assessed with scores of 2. Other detailed information from each trial was also listed in Table S1.

### Total effect of efficacy

**Figure 2:**
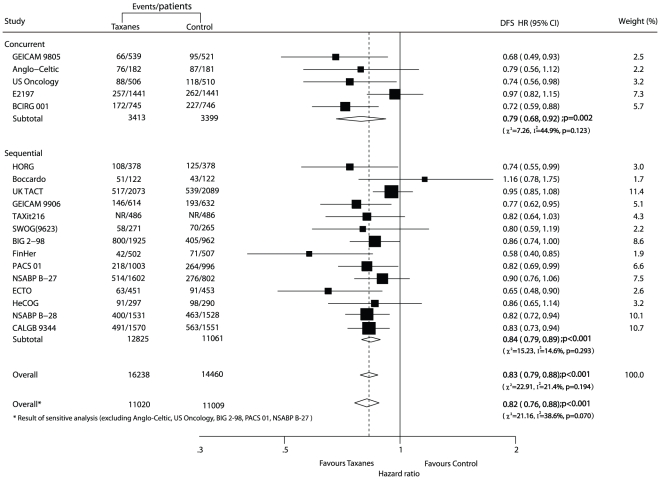
Taxane-based therapy versus non-taxane-based therapy: meta-analysis of disease-free survival (DFS). NR: not report.

Data for DFS were available from all 19 trials [20]–[25], [27], [29]–[30], [32]–[38], [40]–[42] with 8426 events reported. The taxane-based treatment arm was associated with a clinically and statistically significant 17% improvement in DFS when compared with the taxane-free treatment arm (HR = 0.83, 95% CI 0.79–0.88; p<0.001; Figure 2) under a random-effect model, and there was no evidence of significant heterogeneity among individual trials (p = 0.194, I^2^ = 21.4%). The taxane-based treatment arm had lower risk of recurrence in both concurrent and sequential regimens than the taxane-free treatment arm (p value 0.002 and 0.000, respectively; test for interaction, p = 0.046).

**Figure 3:**
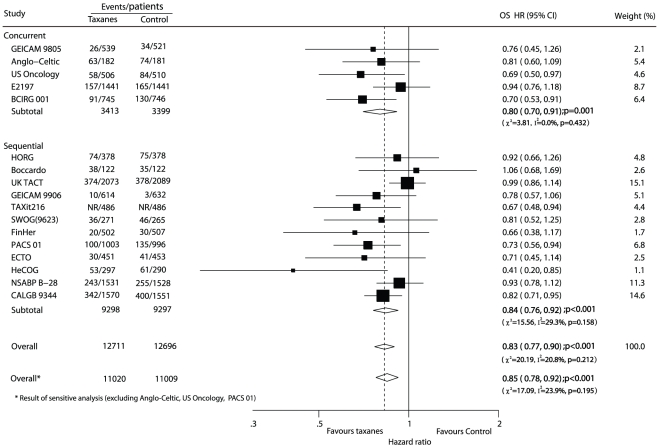
Taxane-based therapy versus non-taxane-based therapy: meta-analysis of overall survival (OS). NR: not report.

OS was reported in 17 trials [20]–[25], [27], [29]–[30], [32], [34]–[35], [37]–[38], [40]–[42] of the 19 trials (BIG 2–98 and NSABP B-27 [33], [36] did not reported OS data), including 25 407 patients who were recruited in the meta-analysis on the risk of death, resulting in 3803 deaths. The efficacy of taxenes on reducing the risk of death was presented more both in all trials (HR of overall 0.83, 95% CI 0.77–0.90) and the trials of different therapy regimens (Figure 3). We found no evidence of publication bias on either DFS or OS by the funnel plots and the Begg-Mazumdar test.

In addition, the sensitivity analysis was conducted among 14 trials [20]–[21], [23]–[24], [27], [29]–[30], [32], [34], [37]–[38], [40]–[42] after excluding 5 trials [22], [26], [33], [35]–[36] with a low Jadad score (score<3). The estimated pooled HRs for DFS (HR 0.82, 95% CI 0.76–0.88) and OS (HR 0.85, 95% CI 0.78–0.92) all favoured the arm treated with taxanes when compared with arm without, and no evidence of significant heterogeneity was observed among individual trials.

### Subgroup analysis of efficacy

#### Node status

**Figure 4:**
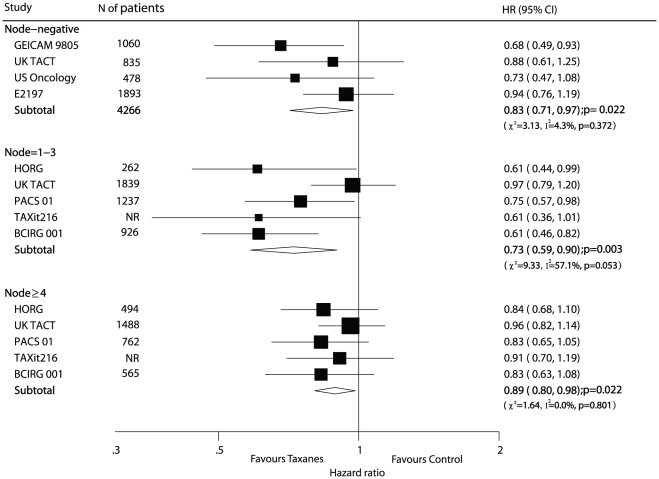
Efficacy of taxanes in subgroup of node-negative, node = 1–3, node 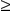
4: meta-analysis of DFS. NR: not report.

Only 4 trials [20], [24], [26]–[27] reported HR for DFS of patients with node-negative BC. The pooled HR of DFS for these trials was 0.83 (95% CI 0.71–0.97, p = 0.022; Figure 4), which corresponds to a 17% reduction in the risk of recurrence among patients with node-negative BC who received taxanes (docetaxel). Among the 19 included trials, 10 trials [21], [23], [29]–[30], [32]–[33], [35], [37], [41]–[42] included only patients with node-positive disease, and the pooled HR of these trials for DFS also favoured taxane treatment (HR 0.82, 95% CI 0.77–0.87; Table 1).

**Table 1:** Taxane-based therapy versus non-taxane-based therapy in subgroups: meta-analysis of disease-free survival (DFS).

	Trials	DFS		p value	Test of Heterogeneity		
		HR	95% CI		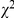	I^2^	p
**Docetaxel**	20,21,22,24,25,27,30,33–37	0.83	0.77 to 0.90	0.000	22.85	43.1%	0.044
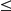 **75 mg/m^2^**	20,22,25,27,33,37	0.81	0.73 to 0.91	0.000	7.63	34.5%	0.178
**100 mg/m2**	21,24,30,34–36	0.84	0.76 to 0.94	0.000	14.98	53.3%	0.036
**Paclitaxel**	23,29,32,38,40–42	0.82	0.76 to 0.88	0.000	5.42	0.0%	0.770
**Weekly sequential**	29	0.77	0.62 to 0.95	0.016	.	.	.
**Every 2 weeks**	32,40	0.84	0.67 to 1.04	0.109	0.10	0.0%	0.752
**Every 3 weeks**	23,38,41,42	0.82	0.73 to 0.93	0.003	4.96	39.5%	0.175
**Trials with N+ only**	21,23,29,30,32,33,35,37,41,42	0.82	0.77 to 0.87	0.000	5.69	23.8%	0.080
**Observation Period**							
**Median Follow–up** 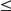 **5 years**	34,35,37,38	0.73	0.64 to 0.83	0.000	5.36	25.3%	0.253
**Median Follow–up >5 years**	20–25,27,29,30,32,33,36,40–42	0.86	0.82 to 0.90	0.000	19.78	24.1%	0.181
**Menopausal Status**							
**Premenopausal**	20,21,23,30,35,37	0.78	0.65 to 0.94	0.010	9.63	48.1%	0.086
**Postmenopausal**	20,21,23,30,35,37	0.78	0.68 to 0.90	0.001	5.47	8.5%	0.362
**ER Status**							
**ER+**	20,21,22,24,27,29,30,35,37,40,41	0.83	0.76 to 0.90	0.000	13.74	27.2%	0.185
**ER−**	20,21,22,24,27,29,30,35,37,40,41	0.80	0.73 to 0.88	0.000	5.88	0.0%	0.826

Furthermore, 5 trials [21], [24], [31], [35], [37] reported HRs for DFS separately in the nodes 1–3 and nodes 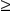
4 subgroups. The pooled HRs also show greater efficacy in the taxane-based treatment arm of the subgroups with nodes 1–3 and nodes 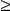
4 (HR 0.73, 95% CI 0.59–0.90, and HR 0.89, 95% CI 0.80–0.98, respectively; Figure 4).

#### Drug dosage, schedule, and observation period

The subgroup analysis of DFS was stratified to trials of different taxane agents (paclitaxel or docetaxel) with different dosage and schedule (docetaxel 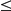
75 mg/m^2^ or = 100 mg/m^2^ and paclitaxel weekly, every 2 weeks, or every 3 weeks) and different observation periods (median follow-up 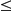
5 years or >5 years). Most of the results showed that the taxane-based treatment arm provided greater efficacy on improving DFS among patients with early or operable BC (Table 1). An 18% HR reduction (95% CI 0.76–0.88) was observed for paclitaxel therapy, a 17% HR reduction (95% CI 0.77–0.90) was observed for docetaxel therapy, and a 14% HR reduction (95% CI 0.82–0.90) was observed in the treatment arm after follow-up of greater than 5 years. Not all taxane schedules might be equal, and table 1 also indicated that there was no significantly statistical difference between the paclitaxel every 2 weeks arm and control arm (HR 0.84, 95% CI 0.67 to 1.04), although analyses of remaining 2 paclitaxel schedules (weekly and every 3 weeks) favoured the taxane-based treatment arm.

#### Others

**Figure 5:**
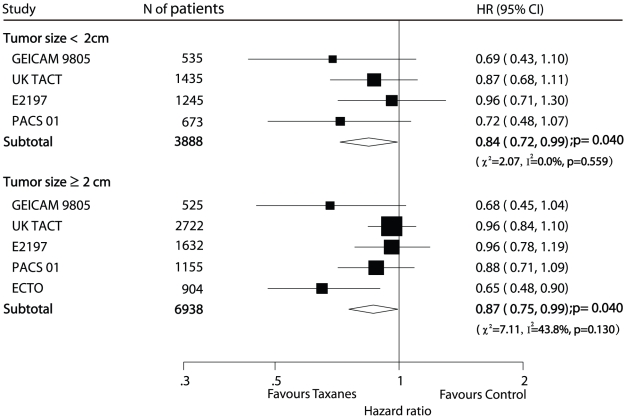
Efficacy of taxanes in subgroups of tumor size <2 cm, tumor size 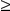
2 cm: meta-analysis of DFS.

**Figure 6:**
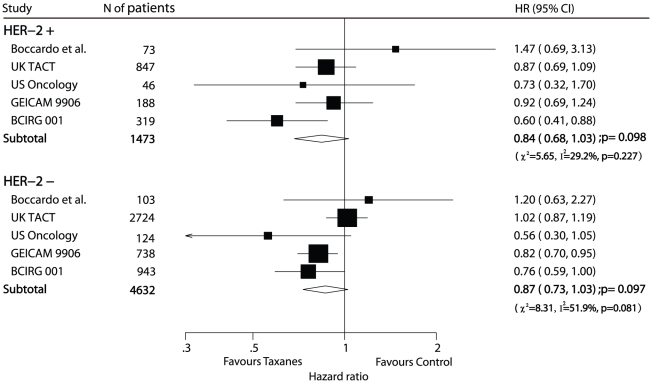
Efficacy of taxanes in subgroups of HER-2 status: meta-analysis of DFS.

Subgroup analysis of patients according to their menopausal status, ER (oestrogen receptor) status, and tumor size was shown in table 1 and figure 5. Superior efficacy of taxanes was found in both premenopausal (HR 0.78, 95% CI 0.65–0.94) and postmenopausal patients (HR 0.78, 95% CI 0.68–0.90) after pooling data from 6 trials [20]–[21], [23], [31], [35], [37]. Efficacy data of adjuvant chemotherapy according to tumer size (<2 cm and 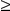
2 cm) was available in 4 trials [20], [24], [27], [35] and 5 trials [20], [24], [27], [35], [38], respectively. The pooled HRs for DFS favoured the taxane-based treatment arm when compared with the taxane-free treatment arm both in the tumor size <2 cm subgroup (HR 0.84, 95% CI 0.72–0.99) and in the tumor size 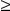
2 cm subgroup (HR 0.87, 95% CI 0.75–0.99) (Figure 5). Eleven trials [20]–[21], [23]–[24], [27], [29], [31], [35], [37], [40]–[41] reported subgroup results of ER status. For ER-positive subgroup the pooled HR of 0.83 (95% CI 0.76–0.90) for DFS indicated a 17% reduction in the risk of recurrence presented in the taxanes-based treatment arm, and for the ER-negative subgroup the pooled HR of 0.80 (95% CI 0.73–0.88) for DFS indicated a 20% reduction in the risk of recurrence presented among patients received taxanes. However, subgroup analysis of HER-2 (human epidermal growth factor receptor-2) status (5 trials [23]–[25], [29], [37] included) showed no significantly statistical difference in efficacy of taxanes when comparing the taxane-based treatment arm with the taxane-free treatment arm in either HER-2-positive group (HR 0.84, 95% CI 0.68–1.03) or HER-2-negative group (HR 0.87, 95% CI 0.73–1.03; Figure 6). Although statistical significance was not attained, when examining the data by HER-2 status, there were similar trends favouring taxanes.

### Toxicities

**Figure 7:**
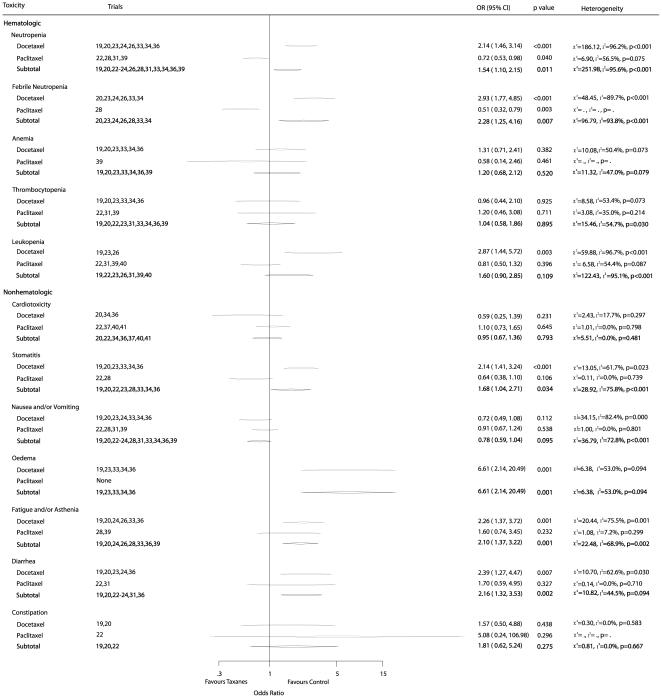
Summary of drug-related toxicities grade 3 or greater.

Data concerning AEs were extracted from 15 trials [20]–[21], [23]–[25], [27], [29], [32], [34]–[35], [37], [39]–[42]. A summary of drug-related toxicities (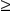
grade 3) was shown in figure 7. The pooled ORs of each group, stratified according to grade 3 or greater toxicities, indicated that a significant increase in toxicity associated with taxane treatment was observed for neutropenia (OR = 1.54, 95%CI 1.10–2.15), febrile neutropenia (OR = 2.28, 95% CI 1.25–4.16), fatigue (OR = 2.10, 95% CI 1.37–3.22), diarrhea (OR = 2.16, 95% CI 1.32–3.53), stomatitis (OR = 1.68, 95% CI 1.04–2.71), and oedema (OR = 6.61, 95%CI 2.14–20.49). However, heterogeneity among trials was found in these analyses, possibly due to the use of different agents at various dosage and the use of different control arms. Moreover, subgroup analyses were performed based on stratification with the 2 types of taxanes. The results suggested that paclitaxel was associated with statistically fewer toxicity events when compared with taxane-free therapy in some toxicities, such as neutropenia (OR = 0.72, 95%CI 0.53–0.98), and febrile neutropenia (OR = 0.51, 95%CI 0.32–0.79) but not in other toxicities. However, figure 7 showed that docetaxel was associated with a significant increase in neutropenia, febrile neutropenia, leukopenia, stomatitis, oedema, fatigue and/or asthenia, and diarrhea (Figure 7).

## Discussion

Nineteen randomized, open-label, phase III trials of 30698 women (with 8426 recurrence events and 3803 deaths) were included to examine the role of taxanes added in adjuvant chemotherapy for patients with early or operable breast cancer. The pooled HRs for DFS and OS for all available trials showed that taxane-based therapy was associated with significant reduction in the risk of recurrence and death, and the similar results were observed in the sensitivity analysis. There was no significant evidence of statistical heterogeneity among individual trials. This meta-analysis also indicated that taxane-based adjuvant chemotherapy was more efficacious in improving DFS and OS when compared with taxane-free therapy. This result was consistent with results reported in 2 previous reviews [6]–[7].

The study conducted by Sparano et al reported that there were no significant differences in DFS between the paclitaxel-treated groups and docetaxel-treated groups [43]. This finding was similar to the results of our study (test for interaction between docetaxel and paclitaxel, p = 0.824) as well as the meta-analysis conducted by De Laurentiis et al (test for interaction between docetaxel and paclitaxel, p = 0.16) [6]. However, Sparano et al also reported that greater benefits in improving DFS were observed in the group receiving paclitaxel weekly and the group receiving docetaxel every 3 weeks when compared with the group receiving paclitaxel every 3 weeks. The results of our subgroup analysis according to the paclitaxel schedule showed that patients receiving paclitaxel weekly and every 3 weeks, but not those receiving paclitaxel every 2 weeks, demonstrated superior efficacy to patients in the control arm of the study. We recognize that comparisons between 2 types of taxanes can be confounded by drug schedule, as shown in the Sparano trial [43]. Because of insufficient data, we were unable to make firm conclusion about the efficacy of various drug schedule (only 4 trials [23], [38], [41]–[42] included in paclitaxel every 3 weeks group and 1 trial [29] included in paclitaxel weekly group).

The results of subgroup analysis also indicated that there were significantly gains in DFS in the taxane-based treatment arm, except for patients with HER-2 status. The pooled HRs of analysis of among women with either HER-2 positive or HER-2 negative status showed a favorable trend but no statistical difference between the 2 treatment arms. However, De Laurentiis et al reported that the HR for DFS in the HER-2 positive subgroup was 0.51 (95% CI 0.29–0.87) and in the HER-2 negative subgroup was 0.70 (95% CI 0.55–0.91) [6]. Only 2 trials [29], [37] were included in their research and the estimated HR may be less reliable. Nowadays, the predictive value of hormone receptor (particularly HER-2) in determining taxane responsiveness remains controversial [44]–[45]. Additional 3 trials [23]–[25] were included in our analysis, and the pooled HR did not confirm the predictive value of HER-2, however, the point estimate of HRs of most trials favoured taxanes. Therefore, the presence of HER-2 as a predictor of taxane responsiveness needs to be further investigated.

A different toxicity profile was confirmed between taxane-based and taxane-free treatment arms. Drug-related toxicities, such as neutropenia, febrile neutropenia, and oedema, were reported in both this study and previous meta-analyses [7]. In our study, the subgroup analyses of toxicity showed paclitaxel may be associated with fewer toxicities than docetaxel. However, this conclusion could not be definitively confirmed because of less available data on paclitaxel. We need more data to support our result in the future. Unfortunately, only 3 of these trials provided information about quality of life (QoL) [20], [37], [40]. These trials showed no significant difference in QoL scores between the 2 treatment arms. Although GEICAM 9805 trial and BCIRG 001 trial [20], [37] found that taxane was associated with a transient reduction in QoL scores, these scores returned to baseline values afterwards.

The efficacy of taxanes for patients with node-negative breast cancer, longer observation periods, and varying tumor size was not reported in the 2 previous meta-analyses. To resolve these uncertainties, we investigated the efficacy by subgroup analysis of available trials.

There was insufficient evidence to define the efficacy of taxanes among the patients with high-risk, node-negative BC, although the efficacy for node-positive, early-stage breast cancer had been confirmed. The benefits of adjuvant chemotherapy (cyclophosphamide combined with methotrexate and 5-fluorouracil) for node-negative disease was confirmed in 3 trials (NSABP B-13, B-19, B-23) [46]. The GEICAM 9805 trial [20] randomly assigned 1060 patients with high-risk, axillary-nod-negative BC to TAC(docetaxel, doxorubicin, and cyclophosphamide) arm or FAC (fluorouracil, doxorubicin, and cyclophosphamide) arm, and the trial reported that the hazard ratio for DFS significantly favoured the TAC arm (HR 0.68, 95% CI 0.49–0.93, p = 0.01). However, 4 trials [24], [26]–[27], [36] reported the efficacy of taxane (docetaxel) for patients with node-negative BC in subgroup analysis. The results of these 4 trials did not show a significant difference between the docetaxel and control arms (the NSABP B-27 trial [36] did not report exact data of HR for DFS in subgroup analysis). Patients in the GEICAM 9805 trial received docetaxel with 6 cycles, and patients in the other 4 trials received docetaxel with 4 cycles. More therapy cycles of docetaxel may be much more beneficial for node-negative BC. Nevertheless, our study did not compare different therapy cycles because of the limited availability of trials. Data concerning DFS from 4 available trials [20], [24], [26]–[27] were pooled (excluded NSABP B-27), and the result (HR 0.83 95% CI 0.71–0.97) significant favored the docetaxel regimen. Therefore, this subgroup analysis provided evidence that docetaxel was useful in improving DFS among patients with high-risk, node-negative BC, which was consistent with the result of the GEICAM 9805 trial.

Trials included in this study were observed with various median follow-up periods. Our aim was to determine whether taxane-based therapy could be efficacious against BC during longer observation periods. The results demonstrated that the benefits of taxanes were still observed during longer follow-up period (HR 0.86, 95% CI 0.82–0.90). However, the results in 2 two trials [22]–[23] (Anglo-Celtic trial and the Boccardo et al. trial with median follow-up of 99 and 102 months, respectively) did not show significant efficacy of taxanes in improving DFS (HR 0.79, 95% CI 0.56–1.12; HR 1.16, 95% CI 0.79–1.75; for these two trials respectively). These results differed from results of our meta-analysis, possibly due to the small sample size of these 2 trials (only 363 patients recruited in Anglo-Celtic trial and 244 patients in Boccardo et al trial). Our study provided stronger evidence demonstrating the efficacy of taxanes for early or operable BC under longer observation periods. Moreover, RCTs which recruit larger population with longer follow-up time will be required to confirm the efficacy of the agent.

The patients included in the ECTO trial [38] all had tumor size greater than 2 cm, with efficacy results showing that the paclitaxel produced significant benefit for this group of patients. For the remaining 4 trials [20], [24], [27], [35], the results did not show any significantly statistical difference between 2 arms in subgroup analysis of tumor size (either tumor size <2 cm or 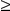
2 cm). However, the estimated HRs using the data of these 5 trials showed taxane-based therapy was statistically superior in reducing the risk of cancer recurrence among patients with both tumor size <2 cm and 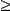
2 cm compared with taxane-free therapy (Figure 5). Consequently, the pooled analysis confirmed the efficacy of taxanes and was consistent with the results of the overall analysis.

Our meta-analysis also has several potential limitations. First, our study was based on abstracted data and not on individual patient data (IPD), which may not provide robust estimation for the association. Second, the characteristics of the included trials were varied in the follow-up observation period, therapy regimen, agents and dosage. Third, there may be several trials with available data that were ongoing or unpublished at the time of the writing of this manuscript that were not included in this meta-analysis, in addition to the 19 trials included in this study. Consequently, publication bias may be unavoidable in this meta-analysis. However, the results form the funnel plots and the Begg-Mazumdar test did not indicate significant publication bias.

Despite the limitations of our research, the results strongly suggest that adjuvant chemotherapy that includs taxanes provides a significant advantage in improving both DFS and OS among patients with early or operable BC compared with therapy without taxanes. Moreover, the subgroup analysis concerning node status also demonstrated that docetaxel-based therapy is superior to docetaxel-free therapy, for high risk node-negative BC, in reducing the risk of cancer recurrence. Additional well-designed RCTs with varying drug schedules for both operable and node-negative BC are warranted to further evaluate these conclusions. The benefit of taxanes should be balanced against their toxicity, and additional data on QoL should be provided in further analysis. Physicians should take these adverse drug events into consideration and interpret the results carefully and comprehensively in clinical practice.

## Supporting Information

Checklist S1 PRISMA Checklist.
(DOC)

Protocol S1 PRISMA Flowchart.
(DOC)

Table S1 Baseline characteristics for included trials.
(DOC)
