# Supplementary material for: Adjuvant Chemotherapy, with or without Taxanes, in Early or Operable Breast Cancer: A Meta-Analysis of 19 Randomized Trials with 30698 Patients
Source: PLoS One. 2011 Nov 1;6(11):e26946. doi: 10.1371/journal.pone.0026946 (PMC3206064; doi:10.1371/journal.pone.0026946)
Supplement: Protocol S1 — PRISMA Flowchart. (DOC) [file pone.0026946.s002.doc]

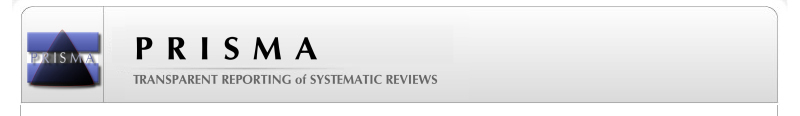
**PRISMA 2009 Flow Diagram**

**Screening**

**Included**

**Eligibility**

**Identification**

Potential articles from PubMed, EMBASE, and the Cochrane (n=4262)

Abstracts and title excluded during first screening (n=5042)

Articles reviewed in details (n=106)

Articles excluded (n=3)

One article without relevant outcomes

One article included locally advanced BC

The data of one article from both arms could not analysed separately

Articles included in meta-analysis (n=23)

Potential articles from meeting and website (n=886)

Articles excluded (n=82)

Reason for exclusion:

Not RCT design (n=26)

Included advanced breast cancer (n=4)

The primary outcome was not DFS or OS(n=26)

Taxane was not the target drug or both arms included taxane (n=26)

Potentially appropriate articles to be included in meta-analysis (n=24)

two additional articles included by reading reviews

19 trials included in 23 articles

Articles with full text (n=18)

Articles with abstract form only (n=5)
