# Supplementary material for: Adjuvant Chemotherapy, with or without Taxanes, in Early or Operable Breast Cancer: A Meta-Analysis of 19 Randomized Trials with 30698 Patients
Source: PLoS One. 2011 Nov 1;6(11):e26946. doi: 10.1371/journal.pone.0026946 (PMC3206064; doi:10.1371/journal.pone.0026946)
Supplement: Table S1 — Baseline characteristics for included trials. (DOC) [file pone.0026946.s003.doc]

Table S1: Baseline characteristics for included trials

| **Trials** | **Year** | **Number of Patients** | **Therapy of Treatment Arm** | **Therapy of Control Arm** | **Regimen** | **Median Follow-up**  **(mouths)** | **Median**  **Age**  **(years)** | **Node Status** | **Main Endpoint** | **Jada Score** |
| --- | --- | --- | --- | --- | --- | --- | --- | --- | --- | --- |
| GEICAM 9805^20^ | 2010 | 1060 | D 75mg/m²,A 50mg/m²and C 500mg/m² day1(p21 days,6 cycles) | F 500mg/m²,A 50mg/m² and C 500mg/m² day1(p21 days,6 cycles) | Concurrent | 77 | 50 | N- | DFS,OS | 3 |
|  |  |  |  |  |  |  | 49 |  |  |  |
| HORG^21^ | 2010 | 756 | D 100 mg/m² p3 weeks(4 cycles) followed by E 75 mg/m² plus C 700 mg/m² p3 weeks(4 cycles) | E 75 mg/m²,C 700 mg/m² and 5-F 700 mg/m² every 3 weeks(6 courses) | Sequential | 62.5 | 56 | N+ | DFS,OS | 3 |
|  |  |  |  |  |  |  | 57 |  |  |  |
| Anglo-Celtic^22^ | 2010 | 363 | A 50 mg/m² and D 75 mg/m² p3 weeks(6 cycles) | A 60 mg/m² and C 600 mg/m² p3 weeks(6 cycles) | Concurrent | 99 | 48 | N+/N- | DFS,OS | 2 |
|  |  |  |  |  |  |  | 49 |  |  |  |
| Boccardo et al.^23^ | 2010 | 244 | P 175 mg/m 2 day p3 weeks(4courses) followed by E 75 mg/m 2 , day 1 and vinorelbine 25 mg/m 2 days 1, 8 p3 weeks(4cycles) | E 100 mg/m 2 day 1 p3 weeks(4 courses) followed by C, M and 5-F (CMF:600, 40, 600 mg/m 2 , days 1, 8) p4 weeks(4 cycless) | Sequential | 102 | 53 | N+ | RFS,OS | 3 |
|  |  |  |  |  |  |  | 54.5 |  |  |  |
| UK TACT^24^ | 2009 | 4162 | F 600 mg/m²,E 60 mg/m², and C 600 mg/m² day 1 p21 days(4 cycles) followed by D 100 mg/m² day 1 p21 days(four cycles) | F 600 mg/m²,E 60 mg/m², and C 600 mg/m² day 1 p21 days(8 cycles) or followed by CMF:C 600 mg/m², M 40 mg/m² F 600 mg/m² days 1 and 8 p28 days(four cycles)/E 100 mg/m² day 1 p21 days(4 cycles) | Sequential | 62 | 48.9 | N+/N- | DFS,OS | 3 |
|  |  |  |  |  |  |  | 48.4 |  |  |  |
| US Oncology^25,26^ | 2009 | 1016 | D 75mg/m² and C 600mg/m² day 1 p21 days(4cycles) | A 60mg/m²and C 600mg/m² day 1 p21 days(4cycles) | Concurrent | 84 | 52 | N+/N- | DFS,OS | 2 |
|  |  |  |  |  |  |  | 51 |  |  |  |
| E2197^27,28^ | 2008 | 2882 | A 60 mg/m² and D 60 mg/m² p3 weeks(4 cycles) | A 60 mg/m² and C 600 mg/m² p3 weeks(4 cycles) | Concurrent | 79.5 | 51 | N+/N- | DFS,OS | 3 |
|  |  |  |  |  |  |  | 51 |  |  |  |
| GEICAM 9906^29^ | 2008 | 1246 | 5-F at 600 mg/m²,E 90 mg/m² and C 600 mg/m² p21 days(4 cycles) followed by eight 1-week courses of P 100 mg/m² 8 weeks | 5-F at 600 mg/m²,E 90 mg/m² and C 600 mg/m² p21 days(6 cycles) | Sequential | 66 | 50 | N+ | DFS,OS | 3 |
|  |  |  |  |  |  |  | 50 |  |  |  |
| TAXit216^30,31^ | 2008 | 972 | E 120 mg/m² d1 q21 (4 cycles) followed by D 100 mg/ m2 d1 q21 (4 cycles),followed by C 600 mg/m² , M 40 mg/m² and F 600 mg/m² d1,8 q28 (4 cycles) | E 120 mg/m² d1 q21 (4 cycles) followed by C 600 mg/m² , M 40 mg/m² and F 600 mg/m² d1,8 q28 (4 cycles) | Sequential | 62 | NR | N+ | DFS,OS | 3 |
|  |  |  |  |  |  |  | NR |  |  |  |
| SWOG(9623)^32^ | 2007 | 536 | A 80 mg/m² days 1, 15, 29;P 200 mg/m² days 43, 57, 71;C 3 g/m2 days 85, 99, 113 | A 80 mg/m² and C 600 mg/m² days 1, 22, 43, 64 followed by high-dose chemotherapy | Sequential | 70 | NR | N+ | DFS,OS | 3 |
|  |  |  |  |  |  |  | NR |  |  |  |
| BIG 2-98^33^ | 2006 | 2887 | Arm II: A75 x 3 followed by D100 x3,followed by CMF x3;Arm III:AD 50/75 x4 followed by CMF x 3 | Arm Ia:A 75 q 3 weeks (4 cycles) followed by CMF (3 cycles) (C 100 day 1-14, F 600 and M 40 d 1+8 q28 day);Ib AC 60/600 (4 cycles) followed by CMF (3 cycles) | Sequential | 62.2 | NR | N+ | DFS,OS | 2 |
|  |  |  |  |  |  |  | NR |  |  |  |
| FinHer^34^ | 2006 | 1010 | D 100mg/m² day 1 p21 day (3 cycles) followed by F 600mg/m²,E 60mg/m² and C 600mg/m² day 1 p21 day (3 cycles) | Vinorelbine 25mg/m² days 1, 8, and 15 p21 day (3 cycles) followed by F 600mg/m²,E 60mg/m² and C 600mg/m² day 1 p21 day (3 cycles) | Sequential | 36 | 50.8 | N+/N- | RFS,OS | 3 |
|  |  |  |  |  |  | 35 | 51 |  |  |  |
| PACS 01^35^ | 2006 | 1999 | F 500 mg/m², E 100 mg/m² and C 500 mg/m² (FEC) day 1p21 days (3 cycles) followed by D 100 mg/m² day 1 p21 days (3 cycles) | F 500 mg/m², E 100 mg/m² and C 500 mg/m² (FEC) day 1p21 days (six cycles) | Sequential | 60 | 50 | N+ | DFS,OS | 2 |
|  |  |  |  |  |  |  | 50 |  |  |  |
| NSABP B-27^36^ | 2006 | 2411 | Arm2:AC(60 and 600mg/m²) p21 days(4 cycles) followed by D 100 mg/m² p21 days(4 cycles ), followed by S;Arm3:AC(60 and 600mg/m²) p21 days(4 cycles) followed by S,followed by D 100 mg/m² p21 days(4 cycles ), | AC(60 and 600mg/m²) p21 days(4 cycles) | Sequential | 77.9 | NR | N+/N- | DFS,OS | 2 |
|  |  |  |  |  |  |  | NR |  |  |  |
| BCIRG 001^37^ | 2005 | 1491 | A 50mg/m²,C 500mg/m² and D 75mg/m² day 1 p21 day (6 cycles) | A 50mg/m²,F 500mg/m² and C 500mg/m² day 1 p21 day (6 cycles) | Concurrent | 55 | 49 | N+ | DFS,OS | 3 |
|  |  |  |  |  |  |  | 49 |  |  |  |
| ECTO^38,39^ | 2005 | 1355 | Arm2:S followed by A 60mg/m² plus P 200mg/m² p21 day (4 cycles), followed by CMF p28 day (4 cycles);Arm3:A 60mg/m² plus P 200mg/m² p21 day (4 cycles) followed by CMF p28 day (4 cycles),followed by S | S followed by A 75mg/m² p21 day (4 cycles) followed by CMF (600mg/m², 40mg/m² and 600mg/2 days 1 and 8) p28 day (4 cycles) | Sequential | 43 | NR | N+/N- | FFP,OS | 3 |
|  |  |  |  |  |  |  | NR |  |  |  |
| HeCOG^40^ | 2005 | 595 | E 110 mg/m² (3 cycles) followed by P 250 mg/m² (3 cycles) and CMF (C 840 mg/m², M 47 mg/m², F 840 mg/m²)(3 cycles) (interval  between cycles was 2 weeks) | E 110 mg/m² (3 cycles) followed by CMF (C 840 mg/m², M 47 mg/m², F 840 mg/m²)(3 cycles) | Sequential | 61.7 | 50 | N+/N- | DFS,OS | 3 |
|  |  |  |  |  |  |  | 50 |  |  |  |
| NSABP B-28^41^ | 2005 | 3060 | A 60 mg/m² and C 600 mg/m² p21 days (four cycles) followed by P 225 mg/m² day 1 (4 cycles) | A 60 mg/m² and C 600 mg/m² p21 days (four cycles) | Sequential | 64.6 | NR | N+ | DFS,OS | 3 |
|  |  |  |  |  |  |  | NR |  |  |  |
| CALGB 9344^42^ | 2003 | 3121 | A(60 ,75 or 90 mg/m²) and C 600 mg/m² p3 weeks (4 cycles) followed by P 175 mg/m² p3 weeks (4 cycles) | A(60 ,75 or 90 mg/m²) and C 600 mg/m² p3 weeks (4 cycles) | Sequential | 69 | NR  NR | N+ | DFS,OS | 3 |

Abbreviations: docetaxel: D; paclitaxel: P; epirubicin: E; doxorubicin: A; cyclphosphamide: C; fluorouracil: F; methotrexate: M; surgery: S; NR: not reported; DFS: disease-free survival; OS: overall survival; RFS: recurrence/relapse-free survival; FFP: freedom from progression; mg/m2:mg per square meter of body-surface area.
